# A transboundary agenda for nature-based solutions across sectors, scales and disciplines: Insights from carbon projects in Southeast Asia

**DOI:** 10.1007/s13280-023-01961-x

**Published:** 2023-12-13

**Authors:** Michelle Ann Miller, David Taylor

**Affiliations:** 1 Asia Research Institute AS8, #07-22, National University of Singapore, 10 Kent Ridge Crescent, Singapore, 119260 Singapore; 2 Department of Geography, National University of Singapore, Singapore, Singapore

**Keywords:** ASEAN, Carbon sinks, Climate change, Nature-based solutions, REDD+, Transboundary governance

## Abstract

Nature-based Solutions (NbS) are integral to efforts to keep global warming below 2°C in accordance with the United Nations’ 2015 Paris Agreement on Climate Change. Yet the transboundary governance dimensions of NbS remain unclear and largely undocumented. In Southeast Asia, NbS have emphasised the conservation and/ or sustainable commodification of carbon sinks found in terrestrial and mangrove forests, seagrass meadows, peatlands and agricultural soils. Mostly project-driven and fixed-term, these “solutions” have often failed to meet their social and ecological objectives. Increasingly, they have added to cross-border problems of: (1) displaced carbon emissions; and (2) economic migration and societal dispossession. This perspective paper delineates a transboundary governance research agenda to mitigate these trade-offs and enhance the co-benefits of NbS in carbon sinks. Building on NbS literature, it identifies cross-sector, multi-scalar and interdisciplinary pathways to improve transboundary cooperation, inclusion and equity in carbon sink governance in varying Southeast Asian contexts.

## Introduction

Recognition that complex environmental problems span local to global scales and affect all people in interconnected (albeit socially and spatially unequal) ways has created a major opportunity for the inclusion of Nature-based Solutions (NbS) in transboundary governance responses. Rising emissions, widespread and profound climate change impacts and global demand for high quality, nature-based carbon credits have highlighted the pressing need for cross-border cooperation in sustaining natural resources (Seddon et al. 2021). As a suite of “actions to protect, sustainably manage and restore natural or modified ecosystems” with societal co-benefits (IUCN 2020, p. 1), NbS could be applied to address these transboundary governance challenges and meet national sustainability goals by reducing emissions and increasing carbon sequestration in plants and soils.

To date, however, work on transboundary environmental governance is not well integrated with related research on NbS. Most nature-based approaches have site-specific “design features that do not easily replicate” (O’Hogain and McCarton 2018, p. 103) and are documented at the project level (Sarira et al. 2022). Since its introduction in 2008 in a World Bank report, the NbS concept has appeared in approximately 700 articles listed in Web of Science and Scopus, with over half of these pertaining to climate adaptation in urban environments, especially regarding flood management (Seddon et al. 2021). Cross-border dynamics are under-theorized and largely undocumented in this literature. However, allied concepts such as ecosystem management, ecosystem-based adaptation and natural capital that have a longer lineage than NbS (Nesshöver et al. 2017; Hanson et al. 2020) have engaged with transboundary forms of governance in specific cases (e.g., building natural capital to sustain common pool resources in the Mekong River Basin, Kura et al. 2017). Transboundary governance variables thus need to be more systematically integrated into nature-based carbon projects that are anchored in localities, which require the coordinated efforts of dispersed actors and networked institutions.

This perspective paper contributes to ongoing efforts to develop holistic approaches to NbS by focusing on their transboundary governance dimensions. We ground our theoretical concern in examples from Southeast Asia, where NbS have increasingly focused on developing the protective or productive functions of carbon sinks (ASEAN 2021), defined as carbon reservoirs and conditions that absorb and store more carbon than they release (UNFCCC, n.d.). Mindful of the contentious (geo)politics surrounding the sink label (Ehrenstein 2018), we do not use this term to subvert local interests to international scientific climate agendas (Seddon et al. 2021) or to assume that ecosystems are “nothing but sinks”, devoid of other values and functions (Kreuter and Lederer 2021, p. 5). Instead, through our introduction of a nature-based transboundary research agenda, we examine the governance dimensions of NbS projects in varying types of carbon sinks relative to their socially and spatially extended co-benefits and trade-offs. Here, it is important to distinguish carbon sinks (ecosystems) from NbS (climate governance strategies). As natural or modified ecosystems, carbon sinks do not constitute governance regimes in their own right. Rather, the materialities they possess make these ecosystems compatible with the broad suite of climate governance principles, actions and arrangements provided by the NbS toolbox.

**Fig. 1:**
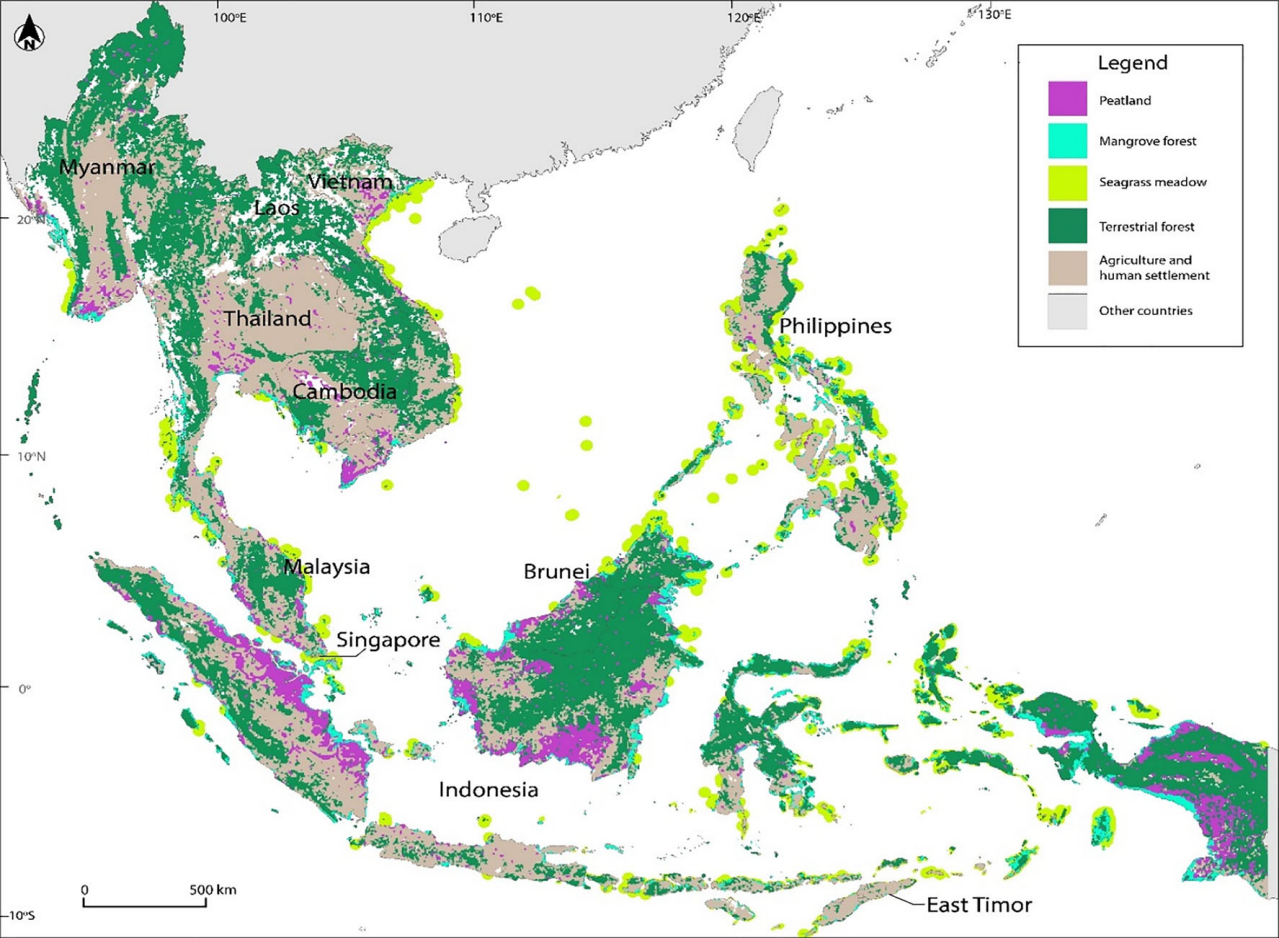
Map showing distribution of main types of nature-based carbon sinks found in Southeast Asia. Adapted from Estoque et al. (2019) and Harris et al. (2021) (for terrestrial forests); Miller and Tonoto (2023) (for mangrove forests); Sudo et al. (2021) (for seagrass meadows); Miller et al. (2022) (for peatlands); and Stibig et al. (2013) (for agricultural soils)

Despite covering only four percent of the world’s land area (Woodruff 2010), the 11 countries of Southeast Asia (Brunei, Cambodia, East Timor, Indonesia, Laos, Malaysia, Myanmar, the Philippines, Singapore, Thailand and Vietnam) have tremendous potential for scalable and cost-efficient NbS actions in carbon sinks (Siman et al. 2021). Southeast Asia’s main types of carbon sinks comprise 196 million hectares (mha) of terrestrial forests (Sarira et al. 2022), 21 mha of peatlands (Ribeiro et al. 2021) and five mha of mangrove forests and seagrass meadows (Fortes et al. 2018; Fig. 1). Partitioned, parceled and often contested, these ecosystems are governed by an extraordinary heterogeneity of fragmented and overlapping land use regimes. The great majority are imminently vulnerable to degradation and denudation, driven by demand for commodities and land for human settlement, industrialization and population growth.

Our concern with the transboundary governance of NbS is bound up in its potential to contribute to whole-of-system transformations that extend beyond carbon sequestration sites and project boundaries. To this end, the following section introduces a transboundary research agenda for analysing the socio-spatial dynamics of NbS in carbon sinks that typically cross jurisdictions and property lines. To organise existing literature and facilitate future research to address these challenges, we propose integrating NbS into three pillars of: (a) cross-sector collaborative relationships; (b) multi-scalar institutional linkages; and (c) interdisciplinary approaches to transboundary governance. We posit that adopting such a holistic transboundary agenda across sectors, scales and disciplines is especially important in Southeast Asia, where the borders of resource organisation have historically been blurred by overlapping formal, informal, religious and customary law (*adat*) systems, traditions and land-use practices (Miller 2022). This argument is developed by exploring pathways for enhancing broadly inclusive cross-border relations to promote plural valuations of nature in carbon sinks and mediate more equitable solutions for distributing co-benefits and trade-offs.

## Nature-based transboundary governance

NbS have attracted strong support in environmental policy circles owing to their relatively low costs compared with many emerging technologies, minimal energy for maintenance, and added value to humanity through their provision of ecosystem services (O’Hogain and McCarton 2018; Osaka et al. 2021). Despite this broad-based appeal, many NbS remain conceptually vague, lacking in legal safeguards and poorly regulated in practice (Cohen-Shacham et al. 2019; Hanson et al. 2020). Diverging understandings of key concepts including “nature” and “solutions” have impeded the transboundary application of NbS (Osaka et al. 2021), reducing their unifying potential to bring together spatially dispersed actors around collectivised forms of carbon stewardship. In ecological terms, ambiguity over such concepts has been shown to generate nature-based carbon projects that inflate sustainability claims by “overselling nature” (Nesshöver et al. 2017, p. 1224) and/or involve the appropriation of nature for private profit (Anguelovski and Corbera 2022). When thus reduced to greenwashing, carbon projects fail to prove additionality, referring to the enhanced sequestration or avoided emissions of carbon. In such cases, NbS could exacerbate rather than reduce transboundary problems of impermanence (future carbon loss) and leakage (displaced emissions to other areas) (Ingalls et al. 2018; Streck 2021). For example, crop displacement in Indonesia is a commonly cited trade-off of forest-based conservation as the conversion of forest lands for agricultural production is shifted to other places (Astuti et al. 2022; Lim et al. 2023). At a broader transboundary scale, the national-level transition in Lao PDR from deforestation to reforestation has displaced deforestation for cultivation to neighbouring countries in the Mekong Subregion (Magliocca et al. 2022). Socially, too, narrowly defined potential solutions may displace local livelihoods, knowledge systems and traditional ways of life, triggering “nature-enabled dispossession” (Anguelovski and Corbera 2022, p. 1). Across agrarian Southeast Asia, the adoption by national governments of European ideas of forest conservation has criminalised traditional customs such as swidden agriculture (controlled land clearing by fire), displacing and dispossessing small farmers onto marginal lands and triggering economic migration that compounds development pressures in other areas (Pichler et al. 2021).

A transboundary approach to NbS could provide some redress for these barriers to effective governance by directing attention to the importance of borders (administrative arrangements) and border relations (strategic processes of coordination and problem-solving to manage cross-border flows) in shaping societal and ecological outcomes (Brunet-Jailly 2022). Transboundary governance is distinguishable from transnational governance in encompassing cross-border relations at sub-national (village, district/municipality, province/state) scales in addition to (supra)national scales (Miller 2020). Governance denotes the institutions and political, economic, legal and cultural dimensions that shape environmental policies and practices, including NbS. Although NbS remains theoretically undeveloped as a tool and focus of governance (Hanson et al. 2020), it has been applied in recent literature to support other key concepts in transboundary governance arrangements. In this supporting role, NbS has been used, albeit with varying definitions, in relation to transboundary species management (López-Cubillos et al. 2022), nature-based tourism (Jones et al. 2021) and various sector-driven conservation actions (Palomo et al. 2021).

To integrate NbS more fully into a transboundary research agenda, we draw from the eight NbS criteria and 24 indicators in the 2020 IUCN Global Standard. We take as our entry point criterion 5 (indicator 5.5), where “the scale of NbS extends beyond jurisdictional boundaries”, necessitating “transboundary cooperation… to enable joint decision-making of the stakeholders in the affected jurisdictions” (IUCN 2020, p. 14). These transboundary governance issues connect with other IUCN criteria concerning appropriately designing the scale of NbS (criterion 2) to address societal challenges (criterion 1) in ways that are adaptive (criterion 7), economically viable (criterion 4), protect biodiversity and ecosystem integrity (criterion 3), equitably balance trade-offs (criterion 6) and sustainably align with existing policy frameworks (criterion 8). Collectively, these intertwined criteria could be flexibly applied to inform NbS activities at meaningful scales and make cross-border collaborative arrangements more adaptive, sustainable and resilient in the longer-term.

Rather than proposing a one-size-fits all transboundary governance framework, we link NbS criteria with recent scholarly efforts to develop the cross-sector (e.g. Raymond et al. 2017; Malekpour et al. 2021), multi-scalar (e.g. Cohen-Shacham et al. 2019; Seymour 2020) and interdisciplinary dimensions of nature-based approaches (e.g. Nesshöver et al. 2017; Hanson et al. 2020). Table 1 outlines actions linking each NbS criterion with these three transboundary dimensions of carbon sequestration projects, which are elaborated in subsequent sections. The NbS action for each transboundary dimension highlights both the “complex intersections of scientific, policy and societal boundaries” and the “need for proactive border crossings” (Petts et al. 2008, p. 599) to encourage the coordination of multiple valuations of nature and cross-jurisdictional solutions at different organisational scales.

**Table 1:** NbS criteria for transboundary governance of carbon sinks

NbS Criterion*		Transboundary applications for carbon sink projects		
		Cross-sector	Multi-scalar	Interdisciplinary
1.	Address societal challenges	Identify and document sector-level priorities in addressing societal challenges (e.g.; food security, fire and flood risks) at planning stage of projects	Document cross-jurisdictional dimensions of shared societal challenges, especially risks and benefits for most proximate users of carbon sink resources	Understand and assess societal challenges from social (e.g.; cultural, political, legal) perspectives in addition to economic and scientific perspectives
2.	Design informed by scale	Map/document how sector-level interventions (e.g.; dyke or road construction) will affect uses of other parts of carbon sink	Upscale investments in pilot projects shown to deliver social and ecological co-benefits (e.g.; sustainable agriculture/aquaculture, eco-tourism)	Whole-of-ecosystem approach needs to combine geospatial data with knowledge of multi-sited human resource connections
3.	Generate net gains for biodiversity and ecosystem integrity	Co-governed NbS interventions by state, private and societal stakeholders can identify, establish and uphold common values of nature	Monitor, assess and incentivize NbS actions that enhance connections between different parts of carbon sink and improve ecosystem connectivity (e.g.; for air, migratory taxa)	Integrate local knowledge of endemic and adaptive species into scientific/ technical approaches (e.g.; dyke construction) to rehabilitate damaged carbon sinks
4.	Economically viable	Account for negative externalities in sector-level assessments of (in)direct costs and benefits of NbS actions in carbon sinks	Connect locally driven NbS contributions to markets and encourage blended (multiple source) financing at different points in supply chains	Supplement economic knowledge of short-term costs and benefits with knowledge of longer-term sustainability measures
5.	Inclusive, transparent, empowering governance processes	Include indigenous and local community stakeholders at centre of co-designed and co-managed partnerships	Establish transboundary cooperation at ecosystem scale in addition to across administrative borders that intersect and divide carbon sinks	Document rights/interests of all stakeholders, especially marginal groups, for plural knowledge production and recourse for future grievances
6.	Equitably balance trade-offs between key goals and multiple benefits	Regulate access to transboundary resources, and safeguard use by most proximate societal users, to ensure trade-offs stay within social-ecological limits	Acknowledge that carbon project trade-offs are transboundary and unavoidable, so must be equitably balanced over time and across jurisdictions	Periodically assess trade-offs to adjust knowledge of compensation and incentives required to protect co-benefits and sustainability goals
7.	Adaptive, evidence-based governance	Flexible cross-sector partnerships increase range of options to respond and adapt to ecosystem transformations, including negative externalities	Regular monitoring and evaluations of NbS actions can identify risks and unexpected events, triggering adaptive responses that can be scaled up or down	Learning based on evidence should include feedback loops, especially from indigenous and local groups, to build adaptive knowledge capacities
8.	Sustainably mainstream in appropriate jurisdictional context	Align cross-sector programs and priorities with (inter)national sustainability frameworks and standards	Scale NbS up (within legal limitations) and out (across jurisdictions and properties) to inclusively retain multiple co-benefits	Integrate intergenerational and cultural knowledge into formal policy choices and legislation

*Adapted from IUCN Global Standard, 2020

These cross-sector, multi-scalar and interdisciplinary pillars of our transboundary agenda for NbS are addressed in turn in the following sections. Taken together, they are used to organise NbS into a conceptual platform that places boundary crossings at the centre of an integrated approach to nature-based governance. When applied to traverse sectoral, scalar and disciplinary boundaries in these ways, we argue that NbS could improve the efficacy and inclusiveness of carbon sink governance in situated Southeast Asian contexts.

## Cross-sector collaborations

### Overview of key challenges

Across carbon sink types, resource governance is heavily specialised and sector-driven. However, sector-level value perspectives of “nature” are only designed to inform “piecemeal interventions” (Cohen-Shacham et al. 2019, p. 27) and partial “solutions” that are offset by continuing trade-offs. This becomes especially problematic in governing resources that flow across property boundaries within and between carbon sinks, such as potable water, clean air and migratory taxa (Palomo et al. 2021; López-Cubillos et al. 2022). Sector-level responses to whole-of-ecosystem problems and societal challenges (e.g., disaster risk reduction and food security) undermine the promise of NbS to deliver multiple co-benefits in the cross-sector policy contexts in which carbon sinks are placed.

When left unresolved, these differences between sectoral interests increase the likelihood of boundary disputes in NbS interventions (Nesshöver et al. 2017). We see this in the United Nations’ REDD+ (Reducing Emissions from Deforestation and forest Degradation) framework, which is widely regarded as a blueprint for future forms of nature-based carbon governance (Kreuter and Lederer 2021). REDD+ has transformed forest governance in many parts of Southeast Asia by implementing NbS such as reforestation, avoided deforestation and community-based agroforestry (Streck 2021). Competing sectoral priorities at the project level have, however, fueled contentious border politics in nature-based carbon projects. In Southeast Asia, REDD+ implementation has often created or exacerbated existing barriers to cooperation at the community level, such as when detractors of outside investments in commercial forest plantations on ancestral lands are pitted against proponents of carbon investments (Astuti and McGregor 2017). These sorts of dynamics, combined with an insufficient understanding of spill-over and displacement effects, have produced sub-optimal societal and environmental outcomes in many REDD+ projects (Streck 2021).

Cross-sector collaborations, defined as the sharing of “information, capacities, resources and decision-making between two or more sectors, in order to achieve a set of outcomes that wouldn’t be achieved separately” (Malekpour et al. 2021, p. 2), may be useful in overcoming challenges that exceed the capacities of individual sectors and groups of stakeholders. To date, NbS research on cross-sector collaborations has not built strong linkages between sectoral interests (e.g., balancing biodiversity with poverty reduction). This is a missed opportunity for holistic forms of cross-border cooperation as different sectors bring discrete knowledge and expertise to bear on multi-faceted environmental and societal challenges that have spatially and temporally cascading consequences (Raymond et al. 2017; Wellmann et al. 2022). Societal groups, especially indigenous communities and agrarian societies, have worked with nature “for millennia” to address transboundary issues of food security and disaster mitigation (Seddon et al. 2021, p. 1521). Yet, increasingly, these place-based communities must rely on scientific advances and private sector funding to adapt to climate uncertainties and shifting markets that affect local livelihoods. The public sector, renowned for its institutional partitioning of interconnected environmental issues (e.g., government agencies that deal separately with mangrove forests and coastal fisheries) has much to learn from the intergenerational knowledge of traditional societies that value human-nature relations within and between jurisdictionally divided land/waterscapes (Miller and Tonoto 2023). In Southeast Asia, governments are also heavily dependent on the private sector to support under-funded programs and build technological and logistical capacities for monitoring, reporting and assessing NbS (Leo 2021). Privately owned agribusinesses (Murdiyarso et al. 2010), smallholdings (Wijedasa et al. 2018) and mining companies (Bauernschuster et al. 2022)—all leading drivers of carbon sink loss in Southeast Asia—continue to grapple with accumulating disaster risks that threaten economic growth and supply chain continuity. As such, they, too, need to seek out avenues for cooperation with government and societal partners to offset these risks by adopting more sustainable development practices using nature-based approaches.

### Transboundary implications for Southeast Asia

In its 2021 Climate Change Report, the intergovernmental union of ten Southeast Asian countries that constitute the Association of Southeast Asian Nations (ASEAN) articulated the need for “a holistic ASEAN regional narrative” (ASEAN 2021, p. 6) to develop “the potential of NbS in all sectors” (*Ibid*, 106) to “not only increase GHG sinks but also provide adaptation co-benefits” (*Ibid*, 119). This region-wide climate strategy arose out of recognition that effectively responding to intensifying and costly environmental threats that bypass the borders of conservation and protected areas and terrestrial-aquatic ecosystems requires cross-border cooperation and buy-in from all sectors. Recent global assessment reports (e.g., the Intergovernmental Panel on Climate Change [IPCC] and the Intergovernmental Science-Policy Platform on Biodiversity and Ecosystem Services [IPBES]) have advocated embedding NbS in multi-sector frameworks for similar reasons (Seymour 2020; Seddon et al. 2021). In unifying the NbS concept into multi-stakeholder regional guidelines (e.g., the ASEAN Multi-Sectoral Framework on Climate Change and Food Security), ASEAN aims to strengthen its member countries’ Nationally Determined Contributions (NDCs) to the Paris Agreement. At present, NbS are so seriously “underrepresented” within NDCs (ASEAN 2020, p. 1) that one study found they only constitute 5.7% of the entire suite of green policy solutions used by ASEAN member countries (Anbumozhi et al. 2022). This situation is not unique to Southeast Asia, as NbS receive less than 3% of climate finance globally (Temasek 2021).

Notwithstanding the funding challenges and operational confusion associated with NbS, there is mainstream appreciation in Southeast Asia for the importance of ecosystem health to economic productivity and human well-being. This widely held value perspective has generated numerous public–private, private-societal and hybrid co-governance environmental partnerships in recent decades (Miller et al. 2020). Table 2 provides examples of nature-based, cross-sector carbon partnerships in Southeast Asia and the border relations that shape their transboundary governance. These cases will be used throughout the remainder of this article to illustrate specific points. Cases were selected based on: (1) their self-articulation of project goals centred on “nature-based solutions”, “natural climate solutions” or “climate mitigation solutions”; (2) their focus on varying types of carbon sinks located in Southeast Asia; and (3) the availability of relevant supporting literature (journal articles, NGO reports, media statements and fact sheets) about individual carbon projects, including their transboundary dimensions. We combined the search terms in criterion 1 with additional search terms in criterion 2 on “blue carbon”, “carbon project/partnership”, “forests”, “mangroves”, “seagrass”, “peatlands”, “agricultural soils/climate agriculture”, “Southeast Asia” and “borders/boundaries”. This initially yielded eleven cases, which we narrowed down to five by only retaining the most extensively documented example for each type of carbon sink (criterion 3). Through these cases, we aim to show diversity in cross-sector carbon projects found across the region.

**Table 2:** Examples of cross-sector collaborations for NbS interventions in carbon sinks of Southeast Asia and the border relations that shape their transboundary governance

Project name/ funder/duration	Cross-sector arrangement by type/key stakeholders	Main NbS intervention/IUCN criterion	Project location* and border relation
Name: Grow Ahead Project Funder: Online donations (crowdfunding) Duration: Ongoing since December 1995	Type: Community-led societal-private partnership	Reforestation of native trees and biodiversity regeneration in integrated farms and community forests (criterion 3) Enhance food sovereignty through inclusive agroecological production (criteria 1, 4, 5, 6) Transmit intergenerational knowledge for adaptive climate capacities (criteria 3, 5, 7)	Project location: AOP operates across 30 provinces in Thailand
	Key stakeholders: Assembly of the Poor (AOP, unregistered Thai community organization) with small farmers, fishers and food producers		Border relation: (a) grassroots networks of subsistence farmers across (sub)national borders; (b) AOP is member of La Via Campesina international peasant organisation in Region of Southeast and East Asia (SEEA LVC)
Name: Oceanus Conservation Mangrove Restoration Project Funder: Online donations Duration: 2022 – 2027	Type: NGO-led multi-stakeholder partnership	Restoration, monitoring and avoided deforestation of mangroves for ecosystem co-benefits (criterion 3) Donations offset individual carbon footprints through mangrove replanting (criterion 4) Assists local government partner aims and nationwide greening programs (criterion 8) Educate, engage and employ community partners in science-based mangrove (re)planting and monitoring (criteria 4, 5, 7)	Project location: 240ha of mangrove forests across provinces of Surigao del Sur, La Union and Misamis Oriental, the Philippines
	Key stakeholders: Oceanus Conservation with Global Landscapes Forum, The Oceancy (sustainable tourism NGO), Synchonicity Earth (UK charity), local governments and mangrove communities in Philippines		Border relation: (a) Mangrove communities liaise with Oceanus, local government units and global and local NGOs; (b) Oceanus promoted through ASEAN-China Mangrove Conservation Network
*Name:* Internationale Klimaschutzinitiative/ International Climate Initiative (IKI) Seagrass Ecosystem Services Project (Southeast Asia component of multi-region project) Funder: Government of Germany (€4,780,000) Duration: 2019–2023	Type: Public-societal partnership	Community-led conservation of seagrass ecosystems and biodiversity (criteria 3, 5) Locally managed marine areas (LMMA) sustain fisheries and ecosystem health (criteria 1, 4, 6) Education for adaptive business models reduces pressure on marine resources (e.g.; spirulina farms, sustainable aquaculture, ecotourism homestays) (criteria 4, 5, 6, 7) Short-term spatial closures replenish fast-recovering marine taxa (criterion 6)	Project location: Multi-sited partnerships in five Southeast Asian Countries (Indonesia, Malaysia, Philippines, Timor-Leste, Thailand)
	Key stakeholders: German government (IKI) with YAPEKA (Indonesian NGO), Marecet (Malaysian NGO), Community Centred Conservation (C3) (Philippines NGO), Save Andaman Network (SAN) (Thai NGO), Blue Ventures (with NGOs in Timor-Leste)		Border relation: (a) Coastal communities with local and international NGOs; (b) implementation partners in Southeast Asia with German government
Name: Korea-Indonesia Forest Management Unit [FMU]/REDD + Joint Project Funding: Korean and Indonesian governments (1st phase), Korindo (2nd phase) Duration: 2013- 2015; follow-up monitoring: June 2019 – May 2020	Type: Bilateral multi-stakeholder partnership, implementation led by private company	Increased fire-fighting capacities FMU in unlicensed plantations brings wildfire/ air pollution reduction and biodiversity co-benefits (criteria 1, 3, 7) Aligns with national zero-burning policy in peatlands and international transboundary haze pollution legislation (criterion 8)	Project location: 14,743ha of unlicensed/ illegal peatland plantations in Kampar Peninsula, Riau, Indonesia
	Key stakeholders: Governments of South Korea and Indonesia, Korindo agribusiness, FMU (Forest Management Units), peatland communities		Border relation: (a) FMU liaise with surrounding peatland communities and local government agencies; (b) South Korean and Indonesian government partnership; (c) Korindo consultancy in Jakarta coordinates implementation with sub-national government agencies
Name: EFICAS (Eco-Friendly Intensification and Climate Friendly Ecosystems) Project Funding: (1) EU under Lao PDR Government’s Climate Change Alliance Program; (2) French Development Agency Duration: 2014 –2019	Type: Public sector-led multi-stakeholder partnership	Agroecological intercropping regenerates soil-based carbon and biodiversity (criterion 3) Engaging farming communities in participatory planning and implementation of climate-resilient agricultural transition (criterion 5, 8) Improve human nutritional status while maintaining soil fertility (criteria 1, 3) Builds adaptive capacities to cope with external stressors and shocks (criteria 1, 7)	Project location: Five provinces (Houaphan, Luang Prabang, Phongsaly, Sayabouri, Xieng Khouang) in Northern Laos
	Key stakeholders: Lao small farmers and their communities, Lao DPR Government agencies, EU, FDA, CIRA, Agrisud		Border relation: (a) Government of Lao DPR liaises with international funders; (b) Lao national government and five provincial governments; (c) Provincial coordinator and Village Land Management Committees (VLMC)

*Project locations are only approximate due to the limited availability in the public realm of data on the precise location of project boundaries. This ambiguity may have important consequences for border relations, especially in remote and rural areas where project boundaries may in reality appear arbitrary

Collectively, these examples reflect the fixed duration of the majority of NbS, especially REDD+ projects, that are often criticised for their short-term financing by a limited pool of donors (Ladekjær Gravesen and Funder 2021). But they do not represent the global or regional land/waterscape of NbS in two interconnected ways. First, the global focus of nature-based projects is on terrestrial forests rather than on other types of carbon sinks (Seddon et al. 2019). A systematic review of recently published literature relating to the governance of carbon sinks in Southeast Asia similarly showed that terrestrial forests were the focus of 84% of 94 selected articles, followed by peatlands and agricultural soils (21%) and mangrove forests and seagrass meadows (13%) (Liu et al. 2022). Second, owing to this prevailing emphasis on terrestrial forest sinks, most NbS involving carbon sinks are enacted within REDD+ frameworks (*Ibid*). In reality, a range of nature-based interventions involving carbon sinks lie outside the purview of REDD+ (Table 2).

The border relations outlined in Table 2 generate different co-benefits and trade-offs at varying organisational scales of governance (see also Table 3). Some leakage is unavoidable in all emission reduction projects (Kreuter and Lederer 2021). Societal displacement, livelihood dispossession and economic migration also frequently follow carbon-based conservation schemes that exclude sustainable food production and local resource rights (Sovacool et al. 2021). Despite their prevalence, these cross-border impacts are rarely factored into the design or planning of NbS. Holistic approaches to the governance of nature-based carbon projects need to better account for the role of border relations in shaping real world connections between spatially and temporally dispersed human-nature connections and to anticipate results that cannot be easily contained or counted within project boundaries.

**Table 3:** Horizontal and vertical scales of transboundary co-benefits and risks in nature-based carbon projects of Southeast Asia

Horizonal/ spatial scale		Vertical/ hierarchical scale			
Project/ boundary approach		Local community	Sub-national (municipality, state, province)	National	Regional/ASEAN
Landscape/ ecological	Oceanus Conservation Mangrove Project	Co-benefit: Landscape approach to reforestation enhances health of interconnected coastal ecosystem services and functions; sustainable livelihood opportunities reduce out-migration imperative	Co-benefit: Memorandum of Understanding between local government units and mangrove communities enhances multi-stakeholder commitment to biodiversity through coordinated action plan	Co-benefit: Aligns with Philippines’ Nationally Determined Contributions (NDCs), national development plans and two national blue carbon committees that all aim to harness potential of blue carbon for climate adaptation	Co-benefit: Project included in planned ASEAN-China mangrove eco-corridor along land-sea trade route that aims to upscale blue carbon opportunities and ecosystem co-benefits
		Risk: Heightened resource insecurity owing to increased encroachment of economic interests (e.g.; mangrove charcoal and timber producers, over-fishing)	Risk: Environmentally friendly and sustainable forms of development may be forfeited to economic imperatives, perpetuating leakage and impermanence from felled mangroves	Risk: Demand may exceed sustainable supply of nature-based carbon credits	Risk: Region-wide resource protections may be lost as port facilities/ coastal development expands within China’s Belt and Road Initiative (BRI) for trade and transport connectivity
	IKI Seagrass Ecosystem Services Project	Co-benefit: Community-led conservation safeguards food security, sustainable livelihoods and climate resilience in coastal ecosystems, limiting out-migration and emissions	Co-benefit: Partnering with local NGOs and coastal communities, Local Governments for Sustainability (ICLEI) acquire ecological knowledge and build capacities to implement and scale up NbS activities in connected coastal-terrestrial ecosystems	Co-benefit: LMMAs are seen by national governments in project countries as sustainable, scalable and cost-efficient blue carbon sinks	Co-benefit: Aligns with ASEAN agenda to expand marine protected areas threefold for region-wide climate adaptation
		Risk: Deteriorating biodiversity and ecosystem health as marine protected areas attract outside eco-tourists (increasing risk of reef trampling) and poachers	Risk: Economic displacement of sustainable business models (including coastal community narratives on seagrass and dugong conservation)	Risk: Climate change and development pressures (e.g. over-fishing, pollutants, dredging, coastal development) risk degrading marine and ecosystem health	Risk: Weak coastal law enforcement increases risk of poachers, pollution and unrestrained coastal development, compromising durability of blue carbon projects
Jurisdictional	Grow Ahead Project	Co-benefit: Provides local food security; protects biodiversity and communities against evictions that displace land use pressures	Co-benefit: Networked linkages between small and landless farmers across jurisdictions foster knowledge exchange about alternative agriculture based on NbS principles instead of cash crop cultivation	Co-benefit: Nationwide grassroots network supports subsistence farming communities in building integrated and sustainable agroforestry capacities	Co-benefit: AOP’s integration into Southeast Asian chapter of global La Via Campesina peasant movement bolsters struggle for food sovereignty and peasant rights to protect nature against global capitalism
		Risk: Lack of legal protections reinforce small farmer vulnerability to capture by outside developers, risking gains in sustainable livelihood transitions and rural poverty alleviation	Risk: Historical antagonism between AOP and Thai State creates barriers to cooperation with local governments and upscaling sustainable NbS	Risk: Small-farmer resource rights and tenurial security may be forfeited to state, military and privately owned agroforestry companies	Risk: AOP’s exclusion from ASEAN frameworks makes it difficult to upscale grassroots-led NbS innovations
Mixed jurisdictional-landscape	EFICAS	Co-benefit: Village Land Management Committee (VLMC) fostered multi-stakeholder cooperation to manage flows between agrarian system components using natural materials	Co-benefit: Feedback loop of project targeted district and provincial-level government authorities to draw lessons for scaling up NbS actions	Co-benefit: Laos government framed EFICAS within NDCs and Global Climate Change Alliance (GCCA) program to build institutional capacities and policy advice at (sub-)national scales	Co-benefit: Regional diffusion of agroecological practices can improve region-wide management of emissions and sustain forests’ climate regulation services
		Risk: Leakage at faster rate; lower crop yields owing to livestock crossing property boundaries; unsustainable cultivation amidst agricultural intensification	Risk: Existing conservation efforts and reforestation programs may sequester less carbon as cultivation expands into neighbouring jurisdictions	Risk: Project problems risk being replicated in other areas if NbS scaled up/ out without first providing redress for shortcomings	Risk: EFICAS may not provide regional-level model of best practice if promoted to ASEAN without first addressing negative externalities
	Korea-Indonesia FMU/REDD + Joint Project	Co-benefit: Increased fire-fighting capacities in peatland communities reduced risk of biomass wildfires that cause carbon loss and transboundary air pollution	Co-benefit: Local government coordination with FMU ensured standardised implementation of REDD + programs	Co-benefit: Top-down approach to REDD + aimed to ensure consistency in objectives and coherent implementation across project areas nationwide	Co-benefit: South Korea increased geopolitical leverage in ASEAN through country-level REDD + interventions
		Risk: Outsourcing of consultancy services may replace sustainable forms of community-level engagement	Risk: Reduced provincial-level capacity building if local government lacks control over centrally controlled and funded project, incubating impermanence	Risk: National emissions may not be well regulated if large polluting businesses are not held legally accountable	Risk: If South Korea’s green soft power is used to support overseas Korean business interests rather than place-based conservation and local livelihoods, unsustainable bilateral partnerships may ensue

A starting point in this pursuit would be to spatially demarcate the range of sectoral interests that underpin border relations in carbon projects (Zingraff-Hamed et al. 2020). In Southeast Asia, where informal or *adat* border relations between traditional communities pre-date and/or co-exist alongside formal governance arrangements, there is an urgent need to clarify boundaries of resource organisation (Miller 2022). Consensual boundary setting practices, such as participatory mapping methods, are vital for spatially representing all sectoral interests and land/water use activities in and around carbon sinks (Astuti et al. 2022), particularly those of indigenous stakeholders and local communities. For instance, YAPEKA (*Yayasan Pemberdayaan Masyarakat dan Pendidikan Konservasi Alam*, Foundation for Community Empowerment and Nature Conservation Education), the Indonesian NGO partner in the German-funded Internationale Klimaschutzinitiative (IKI—International Climate Initiative) Seagrass Ecosystem Services Project that prioritises “nature-based solutions for climate change mitigation and adaptation” (GIZ 2020, p. 26), consults coastal communities when compiling data about terrestrial-coastal ecosystem boundaries (Adaptation Fund 2022). With its dual priorities of community empowerment and conservation, YAPEKA treats participatory mapping as a necessary precondition to monitoring and replenishing seagrass ecosystem services for sustainable coastal resource management (YAPEKA 2015). Conversely, excluding indigenous knowledge of natural ecosystem boundaries that establish place-based connections between land and aquatic environments may compromise the goals of nature-based carbon projects at the initial planning stage (Germond-Duret 2022).

Integrating under-represented indigenous and local community valuations of nature into NbS creates new possibilities for sustainably transforming cross-sector partnerships that are iterative and relational (Zafra-Calvo et al. 2020; Nelson et al. 2020). In carbon projects, this recognition of areas of strategic compatibility between sector-level interests carries transboundary governance implications. We see this in the Philippines-based Oceanus Conservation Project that is funded by eco-concerned donors seeking to invest in “nature-based solutions to mitigate climate change” through the “conservation and restoration of blue carbon habitats” (Climate Reality Project Philippines 2022). The mangrove communities and local government authorities in partnership with Oceanus must remain tactically and ideologically open to market valuations of blue carbon, both to implement donor-prescribed NbS within project boundaries and to prevent livelihood capture by outside development interests that could threaten the continuation of donor funding.

Ultimately, the success of NbS depends on cross-sector commitments to prioritise societal and ecological sustainability over solely utilitarian values of nature. Unless carbon projects are premised on multi-sector consensus about the value of public environmental goods and services, then privatised interventions into nature will accelerate spillover effects. In the EFICAS (Eco-Friendly Intensification and Climate Friendly Ecosystems) Project in northern Laos, utilitarian values prevailed when certain groups of farmers seeking to increase crop yields expanded cultivation beyond project boundaries, to the ecological detriment of surrounding forests (EFICAS, n.d.). This shows that NbS such as afforestation or reforestation may not succeed if they are seen as less financially attractive to farmers than commercial crops. Somewhat differently, in Thailand, the Grow Ahead Project’s ambition to “deliver comprehensive climate mitigation solutions to smallholder farmers through targeted agroforestry efforts” (Fairtrade International 2021) has been under continual threat of encroachment by state, military and privately owned agricultural/ commercial forestry corporations in the absence of enforceable environmental legislation. This suggests a need for cross-sector coordinating institutions and regulatory mechanisms to protect the boundaries of nature-based carbon projects from predatory development interests at relevant scales of governance.

## Multi-scalar governance dimensions

### Overview of key challenges

Decisions about the scale of NbS are highly political with cascading material effects that reinforce the need for transboundary institutional frameworks (Seymour et al. 2022). Governments and private companies, and, to a lesser extent, societal groups, invoke scalar politics to delimit the boundaries of their own responsibility for emissions, explain environmental transformations that fall outside their jurisdictions or fields of expertise, and absolve responsibility for carbon leakage to other areas (Ingalls et al. 2018). Although key to understanding how transboundary governance systems operate, these (inter)scalar dynamics of NbS remain understudied. Nascent efforts are underway, however, to upscale the structures and processes of NbS in governance arrangements and to improve understanding of cross-scale linkages (Cohen-Shacham et al. 2019; Seymour 2020).

Our central concern, with cases “where the scale of the NbS extends beyond jurisdictional boundaries” (IUCN 2020, criterion 5.5), has vertical (hierarchical) and horizontal (spatial) scalar dimensions. Vertically, NbS link place-based communities across jurisdictional borders with (sub)national government agendas (such as EFICAS), voluntary carbon markets (Oceanus Conservation Mangrove Restoration Project), transboundary environmental publics (IKI Seagrass Ecosystem Services and Grow Ahead projects) and (inter)national regulatory frameworks (Korea-Indonesia Forest Management Unit [FMU]/REDD+ Joint Project). Horizontally, too, they work across (sub)national administrative boundaries (Grow Ahead Project), private and communal property boundaries (EFICAS and Korea-Indonesia projects) and natural ecosystem borders (Oceanus and IKI projects).

Vertical, or hierarchical, scales of nature-based carbon governance have mainly been examined at either the local level of project implementation (Sarira et al. 2022) or the supranational scale (Ehrenstein 2018). Project-level studies usually ignore or downplay cross-border variables that are both difficult to manage and to account for in budget line items. Conversely, supranational analyses tend towards “framing-out” (Waller et al. 2020, p. 12) to higher organisational scales, cultivating “optimistic unrealism” among governments and policy makers about the scalability of solutions (Ehrenstein 2018, p. 179). Macro-level approaches also frequently over-simplify or misrepresent transboundary realities linked to competing valuations of carbon sink resources. For instance, Locally Managed Marine Areas (LMMA), like those in the IKI Seagrass Ecosystem Services Project, attract outside poachers as biodiversity increases, compounding governance challenges of funding border patrols (Halik et al. 2018).

Recent studies of horizontal scales of NbS remain divided over the competing merits of drawing project boundaries using a landscape (ecological) approach (Cohen-Shacham et al. 2019) or a jurisdictional (administrative) approach (Seymour 2020). The IUCN standard is non-prescriptive in this regard, only emphasising that project design should be informed by scale (NbS criterion 2) to accommodate “both large-scale and small-scale interventions” (IUCN 2020: 3) and the governance systems within which they operate. In reality, neither of these boundary approaches provides a comprehensive means of mitigating negative externalities linked to carbon projects.

Landscape or ecological approaches, especially those that cover a large spatial area or even adjacent carbon sinks (e.g., where tropical peatlands adjoin mangrove forests), can theoretically reduce the loss of carbon beyond project boundaries. Yet, with over 80 definitions for integrated landscape management, the approach varies tremendously in organisation, implementation and lessons learned (Cohen-Shacham et al. 2019). NbS implemented at the landscape scale also frequently lack coordinating governance instruments across jurisdictional and property boundaries (Albert et al. 2021). Moreover, landscape approaches involving REDD+-funded afforestation/reforestation projects, which have been implemented in large areas of Southeast Asia to generate climate-relevant effects, often deplete existing biodiversity and/ or exclude sustainable uses of resources within project boundaries (Kreuter and Lederer 2021).

Just as NbS activities bordered by landscape approaches can result in “biological successes and social failures” (Christie 2004, p. 155), jurisdictional approaches, which are more likely to emphasise social and economic criteria for success, do not necessarily improve ecological outcomes (Miller et al. 2022). Proponents of jurisdictional approaches view administrative scales as the optimal boundaries for NbS due to their alignment with existing legal, policy and regulatory frameworks (Seymour 2020; see also NbS criterion 8, Table 1). As the nucleus of state economic, political and administrative power at the sub-national scale, district/ municipal and provincial/ state jurisdictions are also usually better networked, funded and legislated than landscape approaches (Miller et al. 2022). However, because jurisdictional approaches introduce artificial barriers (such as dams, roads, fences and different land-use regulations in a single ecosystem) that partition or block the natural flow of resources across land/waterscapes, they may lead to the degradation of certain ecosystem services and functions, setting into motion transboundary effects that remain largely unrecorded.

### Transboundary implications for Southeast Asia

By 2050, ASEAN aims to “scale up nature-based solution[s] for enhancing carbon sinks in coastal and inland forest[s] and soils” as the cornerstone of its region-wide response to mitigating societal challenges linked to climate change (ASEAN 2021, p. 125). As REDD+ is the prevailing roadmap used globally for upscaling or downscaling carbon sink governance, this regional plan will likely encounter transboundary challenges. Country-level applications of the REDD+ framework have only begun to tackle transboundary problems of leakage, which is measured, but not avoided, in national GHG accounting (Streck 2021). Additionally, the cross-border movements of people whose livelihoods are displaced—both by the drivers of carbon sink loss and by nature-based carbon projects—are treated as secondary to flows of trade and finance among ASEAN member countries and globally (Miller et al. 2020). The direct and indirect drivers of these negative externalities become more difficult to identify the higher and more extensive the scale of governance (Streck 2021).

ASEAN has begun to experiment with nesting different levels of NbS within polycentric (multi-centred) governance arrangements to enhance cross-border coordination at the regional scale (Fasting et al. 2021). Multi-scale and cross-sector collaborations (two features of polycentric governance) that disperse decision-making across multiple organisations and levels could elicit novel NbS networks to address cross-border sustainability challenges (Calliari et al. 2022). Nesting NbS measures into plural policy domains (e.g., poverty alleviation, livelihood creation, biodiversity conservation) can equally address extra-territorial problems that are cross-cutting in nature (Nelson et al. 2020). ASEAN’s Climate Resilience Network holds promise in this respect as a polycentric platform for fostering regional exchange among multiple sectors to initiate wide-ranging climate actions (Fasting et al. 2021). Yet, the Southeast Asia-wide trade-offs identified in Table 3 reflect ASEAN’s geopolitical stance of non-interference in the domestic affairs of its member countries. ASEAN’s non-interventionist approach places limitations on its ability to formulate cross-border responses to protect localised carbon projects from broader environmental risks and impacts (Miller et al. 2020).

Integrated, cross-scale planning could establish clearer linkages between NbS and border areas where political will or formal capacities tend to be lacking and need to be strengthened at specific scales. For instance, the Grow Ahead Project is horizontally networked with the Southeast Asia chapter of the global La Via Campesina peasant organisation, but is excluded from vertical (Thai government and ASEAN) frameworks and decision-making processes (GrowAhead.org 2022). These exclusionary scalar politics reinforce the lived precarity of subsistence farmers enrolled in the Grow Ahead Project by destabilising their place-based ecological knowledge, despite their evident conceptual preparedness to implement NbS. This situation heightens the transboundary risk of societal dispossession and forced migration that foment borderland spaces for capitalist accumulation and environmental deregulation.

Combining landscape and jurisdictional approaches could provide some redress for problems of scalability. Integrating these two boundary approaches may strengthen horizontal connections between the biophysical priorities of carbon sinks (landscape approach) and the dispersed sectoral interests invested in them (jurisdictional approach). As the Korea-Indonesia FMU/REDD+ Joint Project shows, however, even when landscape-jurisdictional approaches are integrated (in this case, by combining peatland hydrological unit boundaries with repossessed illegal plantation boundaries), conflicts of interest can shift emission reduction costs from corporations onto vulnerable people (Miller et al. 2022). The consultancy hired to manage the Korea-Indonesia FMU/REDD+ project was owned by the Korindo agribusiness (Wire 2019; Companies House 2023), adding fuel to accusations by environmental NGOs that Korindo used the carbon project to greenwash its tarnished record of land use violations, human rights abuses and illegal use of fire to clear lands for planting elsewhere in Indonesia (RAN et al. 2018).

Addressing such transboundary governance challenges in NbS interventions therefore requires “forging links between small details and large outcomes” (Boyd 2006, p. 108). To achieve this, scale-dependent NbS activities (Seymour et al. 2022) need to be established and coordinated by boundary or intermediary organisations that align the overall objectives of carbon projects (Veelen 2020). Examples of scale-dependent activities in the Oceanus Conservation Mangrove Restoration project and the IKI Seagrass Ecosystem Services project include local-level actions that adaptively balance sustainable food production with biodiversity. By contrast, at the national and regional scales, decision-makers and institutions in both projects are mainly concerned with carbon accounting and climate adaptation. Boundary organisations could enhance the overall co-benefits of these scale-specific NbS actions by fostering collaborative border relations to mediate the exchange of interdisciplinary and cross-sector knowledge and to bridge gaps in institutional capacities at relevant scales.

## Interdisciplinary approaches

### Overview of key challenges

As a boundary object, the NbS concept is well-suited to synthesizing the core ideas from overlapping fields of disciplinary and sectoral knowledge (e.g., labelling climate solutions as natural entails boundary work, Osaka et al. 2021; see also Hanson at el. 2020). Genuine interdisciplinarity, however, that “analyses, synthesizes and harmonizes links between disciplines into a coordinated and coherent whole” (Choi and Pak 2006, p. 351), is difficult to achieve in practice (Petts et al. 2008). Time-consuming and uncertain, the work of interdisciplinarity requires a level of integration that moves beyond single disciplinary boundaries to generate “new perspectives, theories, concepts and methodologies” that together form a hybrid body of knowledge (Pedersen 2016, p. 4). In nature-based carbon projects, which are often time-constrained, hierarchical and experimental, interdisciplinarity and intergenerational knowledge remain notional to scientific and financialized forms of knowledge. Carbon inequality (the unequal spatial and social distribution of emissions) and climate injustice (energy use benefits enjoyed by some that cause climate burdens to many others) tend to follow such siloed nature-based interventions that overlook socially structured vulnerabilities and inequities (Anguelovski and Corbera 2022). Overcoming existing disciplinary barriers to operationalizing the core ideas of NbS is therefore vital to prevent the concept from becoming “just another ‘green communication tool’” (Nesshöver et al. 2017, p. 1216) that only weakly integrates sustainability strategies into growth-based development models, diluting co-benefits and expanding the scale of transboundary trade-offs.

Although “disciplinary diversity is fundamental to sustainability” (Nelson et al. 2020: 50), interdisciplinary engagements with green governance concepts like NbS are “not necessarily innocent” (Craddock and Hinchliffe 2014, p. 2). Interdisciplinary assemblages often perpetuate exclusionary ways of presenting knowledge in nature-based carbon projects (e.g., by limiting the role of social science knowledge to communicating information about carbon sequestration and emerging technologies) (*Ibid*). Indigenous knowledge, too, which has gained political legitimacy in global climate discourses for its perceived contributions to place-based custodianship of common heritage, has only been symbolically integrated into many carbon projects to fulfil checklist-oriented criteria about co-designed activities (Kreuter and Lederer 2021). When thus applied to devalue particular societal relationships with nature, interdisciplinary knowledge can inform decarbonization policies that amplify rather than ameliorate socio-economic and ecological inequities (Sovacool et al. 2021), factors that have been shown to fuel cross-border flows of precarious labour and emissions (Dasgupta et al. 2005).

### Transboundary implications for Southeast Asia

At the regional level of ASEAN, there is recognition that because “climate change interventions are interdisciplinary and inter-sectoral”, NbS need to be actively “integrated into existing policies, programmes, plans, and procedures” (ASEAN 2021, p. 9). Alongside this broadly inclusive principle is a growing push in Southeast Asia to boost private sector confidence in investing in carbon credits by accelerating NbS using engineering innovations like artificial intelligence, cloud computing and mechanised approaches to climate-friendly agriculture (*Ibid*; Temasek 2021). This trend acknowledges that NbS are themselves vulnerable to climate change, and, as such, could be strengthened by integrating emerging technologies (Wellmann et al. 2022). Although important in their own right, the role of technological tools of NbS in contributing to sustainability transitions should not be overstated. Technologies need to be carefully adapted to suit the socio-spatial realities that decisively influence the governance of nature-based approaches (Jagt et al. 2020).

**Fig. 2:**
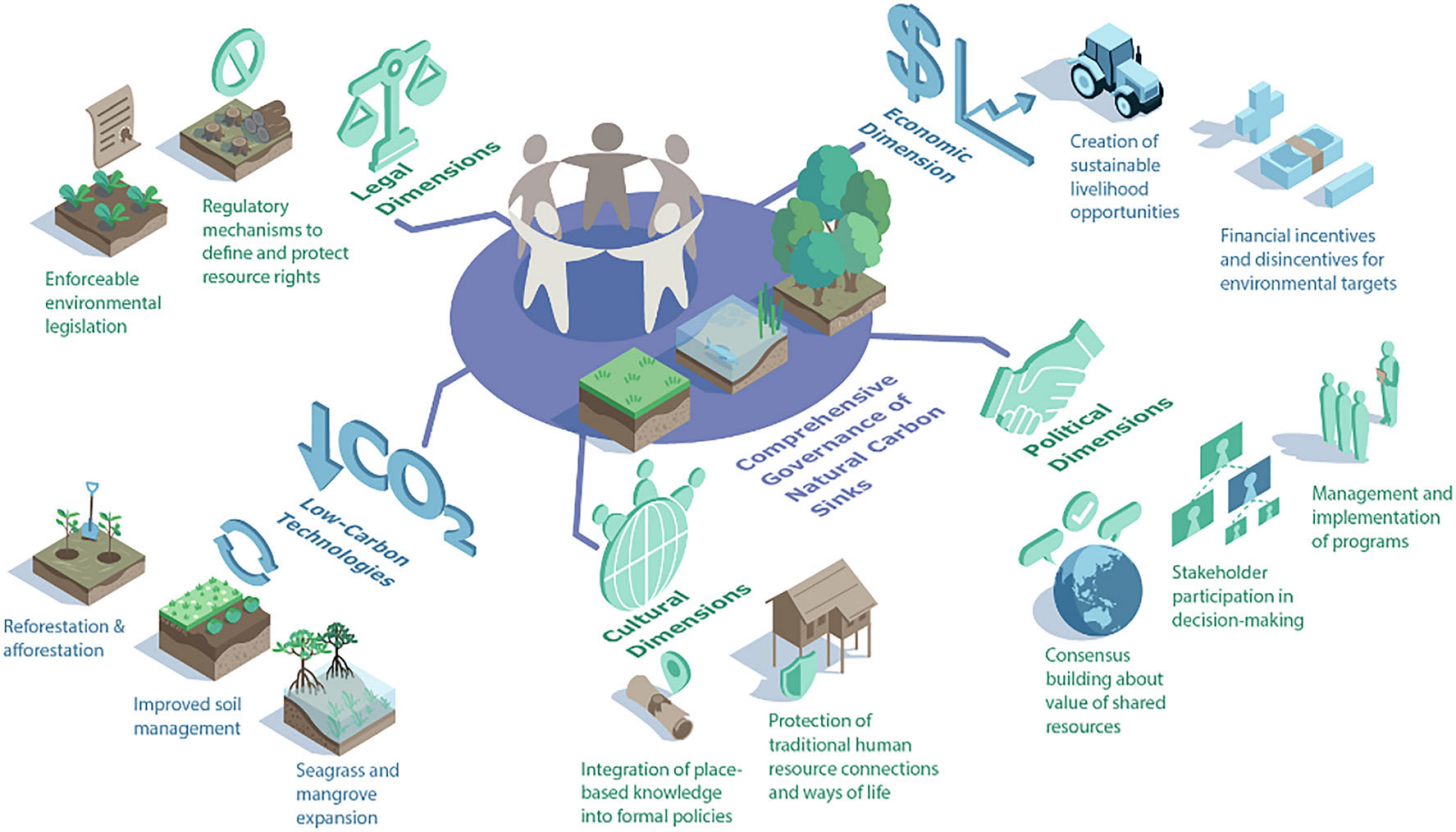
Different disciplinary dimensions that need to be integrated for effective, fair and inclusive carbon sink governance

Figure 2 illustrates how technologies of NbS could be conceptualised alongside equally vital political, economic, legal and cultural aspects of carbon sink governance. It is unproductive to analyse these dimensions separately or within single disciplines as they are relationally intertwined and mutually transformative in practice. We further posit that an interdisciplinary approach to NbS is essential for understanding cross-border issues like competing institutional priorities, power asymmetries and conflicting entitlement claims that fundamentally affect patterns of resource organisation, the uptake of emerging technologies and the scalability of solutions. For instance, in Thailand, antagonistic class relations (political dimension) between the Assembly of the Poor, which coordinates the Grow Ahead Project, and the Thai state, are bound up in competing (non-)monetised valuations of nature (economic dimension) that are rooted in different traditions (cultural dimension) and afforded varying protections (legal dimension) (Missingham 2003; Pye and Chatuthai 2023). These interdisciplinary dimensions permeate all scales of governance. We see this in Southeast Asia’s capital-driven culture that shapes the Thai government’s unwillingness and inability to capture the non-monetised ecosystem co-benefits provided by the Grow Ahead Project’s community-led model of small-scale, self-reliant and ecologically sustainable agroforestry. The portrayal by sections of Thailand’s political and military elite of Grow Ahead as a threat to national development in turn prevents this grassroots project from obtaining registered status, stymieing its scalability and denying its marginalised constituents legal recourse for boundary incursions by outside businesses (Growahead.org 2022).

Interdisciplinary knowledge of these interconnected dimensions could bridge existing barriers to cooperation by creating discursive and physical space for different stakeholders to navigate multiple valuations of nature at varying scales. As a positive example of this, the Oceanus Conservation Mangrove Restoration Project works at the science-policy interface of NbS to integrate place-based knowledge of mangrove communities with scientific monitoring methods that are used to inform and support local and national environmental frameworks and policies (Oceanus 2022). For nature-based carbon projects to succeed in the medium to longer-term, such interdisciplinary knowledge will be critical to collective efforts to adapt to the region-wide impacts of anthropogenic climate change that outpace sectoral and disciplinary understandings. Across much of Southeast Asia, where shortfalls in key areas of state capacity expose carbon sinks to heightened risk of over-exploitation and degradation, interdisciplinary knowledge can help to fill vacuums in formal authority by integrating sector-level valuations of carbon sink resources into multi-sector NbS actions. Without such cross-sector cooperation, border relations are likely to become increasingly conflictual as the demand for carbon sink resources exceeds supply, displacing development pressures and cross-border flows of societal and environmental harm on an ever-expanding scale.

## Conclusion

This perspective paper has made a case for a research agenda to properly account for the transboundary dimensions of NbS in maximising the co-benefits and minimising the trade-offs of carbon projects. These transboundary dimensions require further research into the opportunities for cross-sector collaborations, multi-scalar connections and interdisciplinary approaches to enhance the efficacy and inclusivity of nature-based governance regimes. The study has highlighted how NbS can shape cross-border responses to complex societal and ecological challenges that produce winners and losers at different scales. Drawing from examples in Southeast Asia, we have presented pathways for fostering cross-sector collaborations to address interconnected challenge areas extending across jurisdictions and property boundaries. Fair and inclusive constructions of interdisciplinary knowledge are needed to meaningfully develop these cross-sector and multi-scalar dimensions of NbS in the cross-border policy contexts in which carbon sinks function.

In this pursuit, our transboundary agenda could be applied to guide and underpin future research on:

- Spatial analysis of border relations to account for dispersed sectoral interests;
- Strengthening linkages between discrete sectoral priorities (for example, concurrently optimising sustainable livelihoods and biodiversity);
- Developing the potential of cross-sectoral institutions to function as boundary institutions that align scale-dependent activities with overall project objectives;
- Creating physical and discursive spaces for operationalizing multiple valuations of ecosystem services and functions at relevant scales; and
- Fostering interdisciplinary and cross-sectoral knowledge exchanges around nature-based carbon projects.

Future research on transboundary approaches to NbS might productively integrate older green concepts that have engaged with participatory forms of cross-border environmental governance (Hanson et al. 2020). Accounting for the cross-border variables that shape NbS could provide a firm foundation for planning and managing whole-of-system transformations, as opposed to project-based strategies that mainly seek to optimise carbon capture and storage.

As one such contribution to efforts to facilitate system-level transformations, this study has directed attention towards the importance of accommodating plural perceptions and valuations of nature. Plural perspectives are necessary to strengthen existing NbS arrangements that are both created and reconstituted by a wide variety of transboundary variables. Although the NbS criteria in the IUCN Global Standard are ambitious in scope, real-world applications of nature-based carbon projects continue to downplay both positive and negative externalities. Involving the full range of stakeholders in adaptive planning for cross-border challenges and opportunities as they arise may flexibly alleviate imbalances in the poverty-environment-development nexus that have been shown to accelerate cross-border problems of carbon leakage, livelihood displacement and societal dispossession (Khan 2019).

In Southeast Asia, carbon sinks are unlikely to be long-lasting without targeted NbS interventions to provide a buffer against intensifying development pressures and climatic change. Borders are neither designed nor equipped to withstand these regional and planetary transformations. Carefully factoring border relations into the design of NbS could generate liminal spaces for accommodating multiple valuations of nature, especially indigenous knowledge and marginal voices that tend to be overlooked at higher scales of decision-making. To protect these generative cross-border spaces within the rapidly changing policy environments of Southeast Asia, regulatory safeguards need to be strengthened, both to ensure that NbS are not captured by utilitarian valuations of nature and to rescale successful carbon projects in a just and sustainable manner. Without attention to these issues, bounded carbon projects are unlikely to meet their objectives and may even accelerate lived inequalities and other spillover effects that risk compounding climate impacts on many fronts. 
